# Influence of Substrate Heating and Nitrogen Flow on the Composition, Morphological and Mechanical Properties of SiN*_x_* Coatings Aimed for Joint Replacements

**DOI:** 10.3390/ma10020173

**Published:** 2017-02-13

**Authors:** Charlotte Skjöldebrand, Susann Schmidt, Vicky Vuong, Maria Pettersson, Kathryn Grandfield, Hans Högberg, Håkan Engqvist, Cecilia Persson

**Affiliations:** 1 Materials in Medicine group, Division of Applied Materials Science, Department of Engineering Sciences, Uppsala University, Uppsala 752 37, Sweden; charlotte.skjoldebrand@angstrom.uu.se (C.S.); petterssonemmamaria@gmail.com (M.P.); hakan.engqvist@angstrom.uu.se (H.E.); 2 Thin Film Physics Division, Department of Physics, Chemistry and Biology (IFM), Linköping University, Linköping 581 83, Sweden; sussc@ifm.liu.se (S.S.); hans.hogberg@liu.se (H.H.); 3 Department of Materials Science and Engineering, McMaster University, Hamilton, ON L8S 4L7, Canada; cestlavic@gmail.com (V.V.); kgrandfield@mcmaster.ca (K.G.)

**Keywords:** silicon nitride, coating, hip joint replacement, X-ray photoelectron spectroscopy (XPS), nanoindentation, hardness, Young’s modulus, transmission electron microscopy (TEM)

## Abstract

Silicon nitride (SiN*_x_*) coatings are promising for joint replacement applications due to their high wear resistance and biocompatibility. For such coatings, a higher nitrogen content, obtained through an increased nitrogen gas supply, has been found to be beneficial in terms of a decreased dissolution rate of the coatings. The substrate temperature has also been found to affect the composition as well as the microstructure of similar coatings. The aim of this study was to investigate the effect of the substrate temperature and nitrogen flow on the coating composition, microstructure and mechanical properties. SiN*_x_* coatings were deposited onto CoCrMo discs using reactive high power impulse magnetron sputtering. During deposition, the substrate temperatures were set to 200 °C, 350 °C or 430 °C, with nitrogen-to-argon flow ratios of 0.06, 0.17 or 0.30. Scanning and transmission electron spectroscopy revealed that the coatings were homogenous and amorphous. The coatings displayed a nitrogen content of 23–48 at.% (X-ray photoelectron spectroscopy). The surface roughness was similar to uncoated CoCrMo (*p* = 0.25) (vertical scanning interferometry). The hardness and Young’s modulus, as determined from nanoindentation, scaled with the nitrogen content of the coatings, with the hardness ranging from 12 ± 1 GPa to 26 ± 2 GPa and the Young’s moduli ranging from 173 ± 8 GPa to 293 ± 18 GPa, when the nitrogen content increased from 23% to 48%. The low surface roughness and high nano-hardness are promising for applications exposed to wear, such as joint implants.

## 1. Introduction

Joint replacement surgery is a common procedure that can improve the quality of life for patients suffering from, e.g., arthrosis: in Sweden alone, 16,566 primary total hip arthroplasties (THA) and 13,000 primary total knee arthroplasties (TKA) were performed in 2014 [1,2]. The incidence of both THA and TKA is predicted to increase [3]. The implants are generally considered successful with an average survival rate of 97.8% after three years [4]. Nevertheless, patients are receiving implants at a younger age [5] and living longer and more active lives, which brings on a need for increased implant longevity. Coating metallic components with a ceramic material could improve the surface properties, including wear resistance and biocompatibility, and it could also reduce corrosion and minimize ion release compared to a bulk metal [6]. The metal byproducts have, in some cases, been linked to metallosis, hypersensitivity and pseudotumors [7]. Silicon nitride (SiN*_x_*) has been suggested as a potential coating material [8,9,10]. Bulk silicon nitride (Si_3_N_4_) has previously been used in turbine components and ball bearings and was introduced as a biomedical material for spinal fusion in 2006 [11]. In 2007, silicon nitride was introduced as a potential femoral head material for hip joint implants [12]. It is considered a suitable candidate for joint implant coatings because of its wear resistance [13,14] and dissolution into non-toxic ions in aqueous solutions [15,16,17,18]. Here, a high solubility of the wear particles (decreasing the risk for third-body wear or negative biological reactions), but a low solubility of the coating itself (making it last longer) would be optimal. 

SiN*_x_* and SiN*_x_*C*_y_* have previously been deposited by plasma-enhanced chemical vapor deposition (CVD) and several physical vapor deposition (PVD) techniques [19,20]. The PVD methods used include radio frequency sputtering, direct current magnetron sputtering and high impulse power magnetron sputtering (HiPIMS) [21]. The deposition of SiN*_x_* and SiN*_x_*C*_y_* by HiPIMS has resulted in amorphous sub-stoichiometric coatings with a dense morphology and low surface roughness [10], which could be beneficial in terms of applications exposed to wear. The literature shows that the nitrogen content affects the dissolution rate of SiN*_x_* coatings [17] as well as the hardness (*H*), Young’s modulus (*E*) and surface properties of SiC*_x_*N*_y_* coatings [20,22] and a high nitrogen content has been found to be beneficial in terms of dissolution [17] and a higher hardness and Young’s modulus [23]. Furthermore, the substrate temperature has been found to influence the coating morphology and to introduce a nanocrystalline microstructure for the SiN*_x_* coatings [24]. 

Supported by the above, and from the development of a reactive HiPIMS (rHiPMS) process for SiN*_x_* coatings [23], we herein investigate the effect of substrate heating and nitrogen flow during rHiPIMS of SiN*_x_* coatings on the coatings’ composition, morphology, microstructure, and mechanical properties. 

## 2. Results 

### 2.1. Composition, Microstructure and Nanostructure

The coating composition for the investigated processes, as obtained from X-ray photoelectron spectroscopy (XPS) measurements, is summarized in Table 1. At a substrate temperature of 200 °C the N content increased from 22.8 at.% to 48.0 at.% as the nitrogen-to-argon flow ratio (*ƒ*_N_2_/Ar_) in the plasma increased from 0.06 to 0.30. When the substrate temperature was increased to 350 °C, similar N contents were obtained for the corresponding *ƒ*_N_2_/Ar_, while a further increase to 430 °C resulted in a lower N content (41.3 at.%) at *ƒ*_N_2_/Ar_ = 0.3. The O content was found to range between 1.8 at.% and 5.8 at.%.

**Figure 1:**
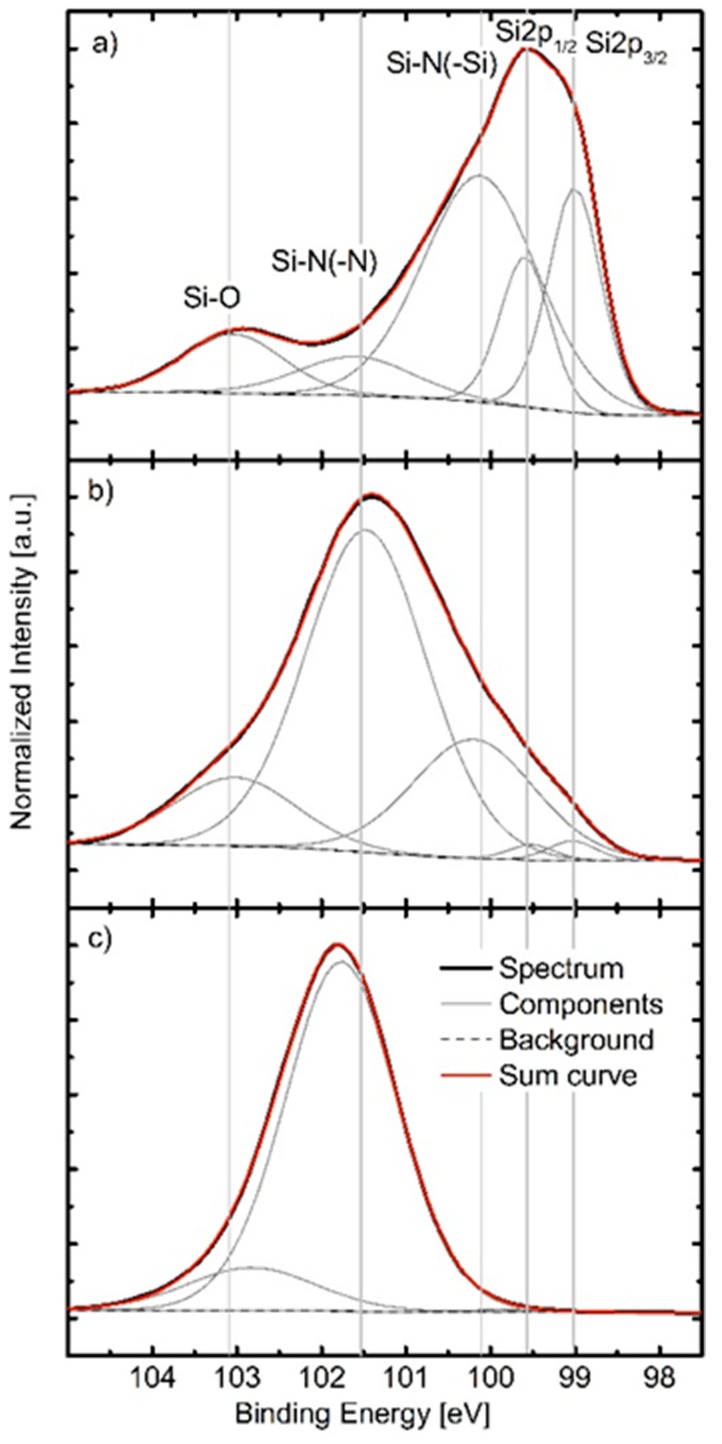
(**a**–**c**) XPS Si2p core-level spectra acquired from coatings deposited using (**a**) process 1; (**b**) process 2 (same behavior seen for processes 4 and 6); and (**c**) process 3 (same behavior seen for process 5). The core-level spectra were acquired on as-deposited samples, without additional Ar^+^ cleaning.

The composition of the coatings was mirrored by their chemical bonding structure. Figure 1a–c show deconvoluted Si2p core-level spectra of processes 1–3. The deconvoluted Si2p core-level spectra of processes 4–6 were omitted as the chemical bond structures of coatings deposited by processes 4, 5 and 6 were similar to the bond structures of coatings prepared by processes 2, 3, and 2, respectively. In Figure 1a–c the different chemical environments of Si in the coating are indicated. The contributions at 99.0 eV and 99.6 eV are assigned to the Si–Si bond doublet, the contribution at ~100.1 eV is assigned to Si–N bonds that are influenced by Si as the next neighbor, at ~101.5 eV a contribution due to Si–N–N or Si–N bonds influenced by N and/or O arises, and between 102.8–103.0 eV the Si–O bond appears. A maximum of five contributions were fitted in spectra of samples deposited by processes 1 and 2 (Figure 1a,b), while spectra recorded for the sample prepared by process 3 only contained two contributions.

**Figure 2:**
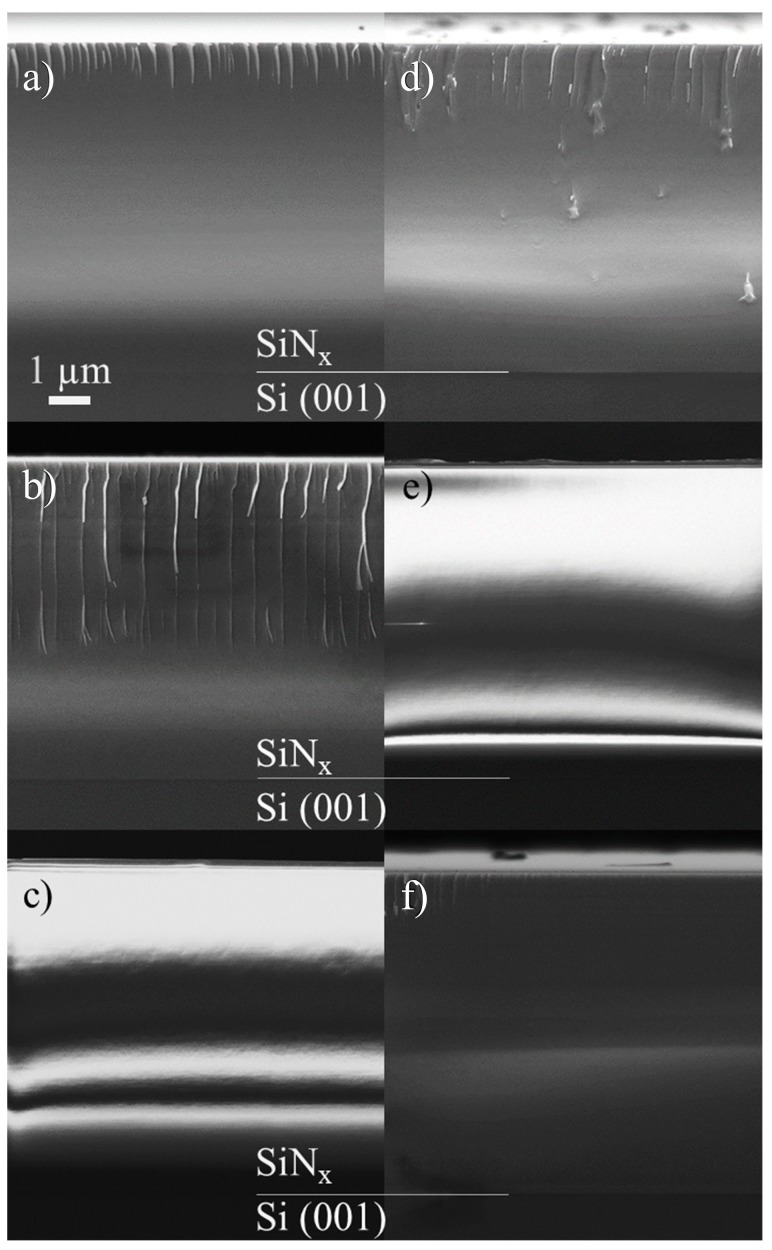
(**a**–**f**) Representative SEM cross-sections of coatings deposited onto Si(001) by (**a**) process 1; (**b**) process 2; (**c**) process 3; (**d**) process 4; (**e**) process 5; and (**f**) process 6. The Si(001)/SiN*_x_* interfaces are indicated.

Scanning electron microscopy (SEM) cross-sections of coatings deposited onto Si(001) are presented in Figure 2a–f. As can be seen in Figure 2a–f, all SiN*_x_* coatings exhibited a featureless, glassy morphology without apparent columns and approximately the same thickness of 7500 nm. Images of coatings with N contents of approximately 48 at.% (Figure 2c,e) displayed charge-up effects due to the increasingly insulating properties of the SiN*_x_*.

Coatings from processes 1, 2 and 3 (all deposited at 200 °C, but with different nitrogen contents) were analyzed using transmission electron microscopy (TEM), and standard FIB procedures were used to isolate cross-sectional lamella [25]. 

**Figure 3:**
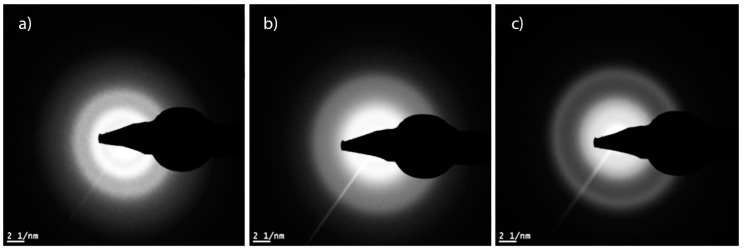
Diffraction patterns from process 1 (**a**); process 2 (**b**) and process 3 (**c**).

Using high-angle annular dark-field (HAADF) TEM imaging, the coatings appeared uniform and amorphous throughout the FIB sample. The diffuse bands in the diffraction patterns suggest that the coatings are amorphous (Figure 3).

### 2.2. Surface Roughness

**Figure 4:**
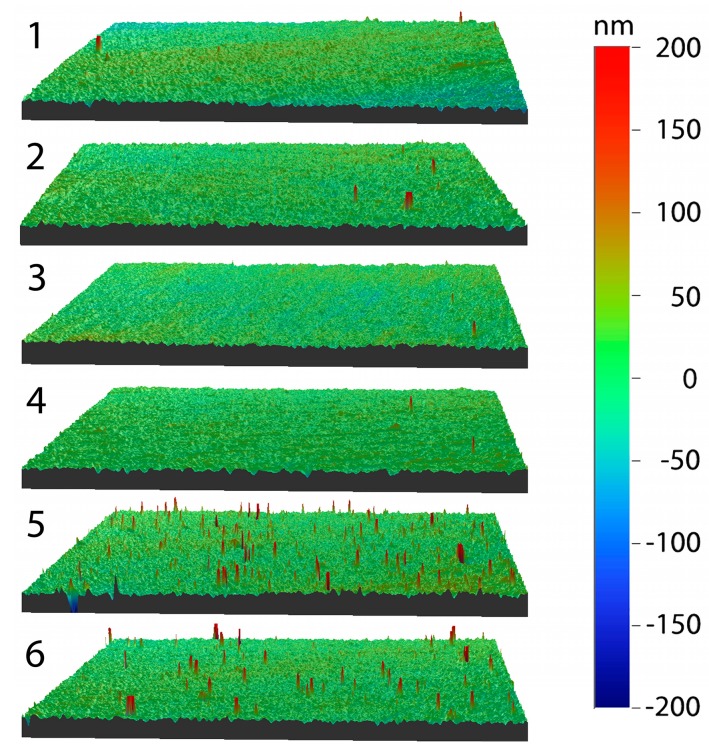
VSI images illustrating the surface for coatings for processes 1–6, the numbers in the figure refer to the process number. The corresponding *R* parameters are found in Table 2.

The arithmetic average surface roughness (*R*_a_) ranged between 10 and 15 nm, i.e., similar to the cobalt chromium molybdenum (CoCrMo) reference (10.7 ± 1.8 nm). Only process 5 showed a significant difference for *R*_a_ (*p* < 0.001) compared to CoCrMo. There was no statistically significant difference in the average roughness between processes with increasing *ƒ*_N_2_/Ar_ (*p* = 0.25) (at the heater power of 1 kW) but a statistically significant effect was observed for an increasing heater power (*p* = 0.03) (for the same *ƒ*_N_2_/Ar_ of 0.30). The increasing *R*_a_ with the substrate temperature was induced by elevated maximum height values (*R*_t_) due to defects, such as droplets or embedded particles. For the mean value of the highest peak-to-valley height (*R*_z_), a significant difference between substrate temperatures 200 °C and 350 °C (*p* < 0.001) and 200 °C and 430 °C (*p* = 0.001) was found. Processes 5 and 6 showed a significant difference of *R*_t_ and *R*_z_ (*p* < 0.001 for both) compared to the CoCrMo reference samples. Examples of the surfaces from each process are illustrated in Figure 4. The surface roughness corresponded to less than 5% of the maximum indentation depth, making the coatings suitable for nanoindentation [26].

### 2.3. Hardness and Young’s Modulus

**Figure 5:**
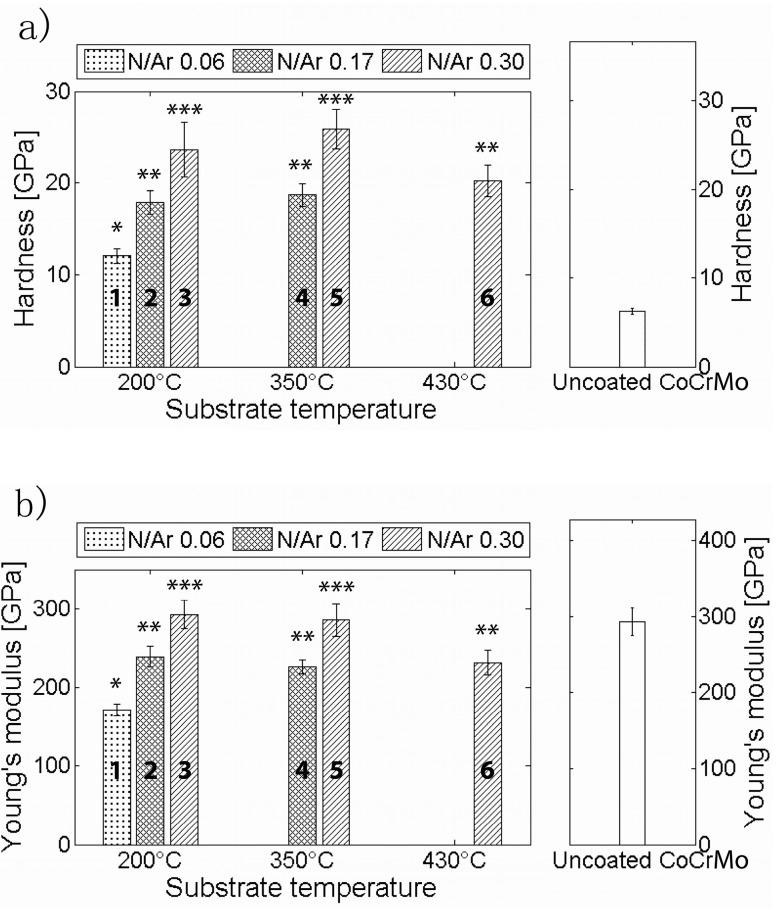
Nano-hardness (**a**) and Young’s modulus (**b**) values as a function of substrate temperature and nitrogen flow ratio. The process numbers are indicated in the graph. Groups that gave significantly different results have been given different letters (*, ** and ***).

The hardness values obtained by nanoindentation are presented in Figure 5 and Table 2. The hardness, as shown in Figure 5a, increased significantly for each increase of *ƒ*_N_2_/Ar_ (*p* < 0.001), which can be seen when comparing process 1 to 2 and 3 or process 4 to 5. It was also significantly higher for the two lowest substrate temperatures (processes 3 and 5) compared to the highest (process 6) (*p* = 0.034 and *p* < 0.001). All coatings exhibited higher hardness values than the CoCrMo reference (6.2 ± 0.4 GPa).

The Young’s moduli (Figure 5 and Table 2) showed the same trend as the hardness: increasing with an increasing *ƒ*_N_2_/Ar_ (*p* < 0.001), and a significant decrease for the substrate temperature between the highest substrate temperature of 430 °C (process 6) compared to temperatures of 200 °C (process 3) and 350 °C (process 5) (*p* ≤ 0.001). The Young’s moduli of all coatings were comparable to or lower than the CoCrMo reference sample (293.1 ± 17.7 GPa).

The hardness and Young’s modulus were related to the nitrogen content and an increase in both parameters was observed. *H*/*E* ratios of the coatings ranged from 0.07 to 0.09. There was a slight increase with the increasing nitrogen content, and the *H*/*E* ratios for all coatings were considerably higher than the ratio for CoCrMo (0.02).

## 3. Discussion

In this study, the effect of substrate heating and nitrogen flow during rHiPIMS was investigated for SiN*_x_* coatings, in terms of coating composition, morphology, microstructure, and mechanical properties. As expected, the nitrogen content increased as the *ƒ*_N_2_/Ar_ increased, from 22.8% to 48.0%, which resulted in coatings with N/Si ratios ranging from 0.32 to 1.05. This is comparable to or higher than previously reported SiN*_x_* coatings with a N/Si ratio of 0.8 [18] and 0.27 to 0.65 [10]. However, the latter study obtained the composition by energy dispersive X-ray spectroscopy (EDX), which could result in lower N/Si ratios as EDX has limitations in terms of its ability to detect light elements. Coatings from process 1 (*ƒ*_N_2_/Ar_ 0.06 and *T* 200 °C) resulted in a high silicon content (72%) and low nitrogen content (23%), i.e., an N/Si ratio of 0.32. This corresponded to bonds of Si–Si type, as determined in the XPS spectrum. Coatings from processes 2 (*ƒ*_N_2_/Ar_ 0.17 and *T* 200 °C), 4 (*ƒ*_N_2_/Ar_ 0.17 and *T* 350 °C) and 6 (*ƒ*_N_2_/Ar_ 0.30 and *T* 450 °C) resulted in silicon contents of 54.6%, 54.4% and 53.9%. While process 6 had a higher N/Ar flow and could be expected to yield a higher nitrogen content, the increase in temperature resulted in a decrease of the nitrogen content. This decrease is likely due to the desorption of nitrogen at the elevated temperature. These coatings displayed an increased influence of Si–N–N bonds, as did coatings from processes 3 (*ƒ*_N_2_/Ar_ 0.30 and *T* 200 °C) and 5 (*ƒ*_N_2_/Ar_ 0.30 and *T* 350 °C). As the nitrogen content increased there was a shift from the Si–N–(Si) bonds towards Si–N–(N). Regardless of the composition and bonding, all coatings were amorphous, as could be seen in the diffraction patterns of the TEM samples. The distances of the rings could not be measured due to their diffuse appearance. This is in agreement with previous studies by Olofsson et al. [8] and Wang et al. [20] which showed similar SiN*_x_* and SiC*_x_*N*_y_* coatings to be amorphous. There was a clear correlation between *ƒ*_N_2_/Ar_ and the mechanical properties, *H* and *E*. Both properties increased with the increasing *ƒ*_N_2_/Ar_ (and hence the increasing N content), an effect believed to be caused by the change in the chemical bonds. The effect of the nitrogen content on the nano-hardness of SiC*_x_*N*_y_* coatings has been demonstrated previously by Wang et al. [20]. They found a maximum nano-hardness for 15.7 at.% nitrogen content and decreasing nano-hardness values for SiC*_x_*N*_y_* coatings with increasing N contents up to 25 at.%. However, the decrease in the nano-hardness of the SiC*_x_*N*_y_* coatings was attributed to an increase of the C–C bond prevalence according to the authors. The nano-hardness values found here agree well with previous studies on SiN*_x_* coatings [8,10], as do the Young’s moduli [10]. The *H*/*E* ratio has been suggested to be a better indicator for wear resistance than the hardness alone, being indirectly related to the elastic strain limit, but also increasing with a higher hardness (resistance to plastic deformation) and a lower modulus (likely to reduce the interfacial stresses between the coating and substrate) [27]. The *H*/*E* ratios of the coatings are similar to previous coatings that showed a low specific wear rate, both similar silicon nitride–based coatings [9] and other coatings such as diamond like carbon (DLC) (*H*/*E* of 0.08–0.12 [28] and 0.11 [29]), hence the investigated coatings are believed to be promising in applications exposed to wear. As previously mentioned, coatings with a high nitrogen content have also been found to yield a lower dissolution rate compared to those with a lower nitrogen content [17]. The mechanical properties, possibly indicating a high wear resistance, in combination with the possibility of a low dissolution rate are promising pointers towards well-performing coatings with high longevity and resistance to wear. A critical aspect that is not covered in this study is the adhesion and wear of the coatings which will be addressed in future studies. 

The substrate temperature had a small influence on the composition. The highest substrate temperature of 430 °C gave a 6.7% decrease in nitrogen content to 41.3 at.% compared to 48.0 at.% for process 3. This was caused by the desorption of nitrogen-containing species at the substrate surface, facilitated by an increased in the ad-atom mobility due to the elevated substrate temperature. Similar results were reported by Musil [30]. The morphology of the coatings, as observed in the SEM cross-sections, was dense and uniform without any texture for all coatings; however there were protrusions on the surface. These defects were mainly caused by arcing [31]. While the *R*_a_ values showed a low variation, the *R*_t_ and *R*_z_ for processes 5 and 6 were significantly higher than for the other processes, which was a result of an increased amount of defects. The mechanical properties decreased slightly for the highest substrate temperature (with a decreased N content). 

## 4. Materials and Methods

### 4.1. Materials and Coating Deposition

The SiN*_x_* coatings were deposited in an industrial deposition system (CemeCon AG, Würselen, Germany). The coatings were grown on CoCrMo discs (ASTM F799, Peter Brehm GmbH, Weisendorf, Germany) and Si(001) wafers, using rHiPIMS processes in a N_2_/Ar atmosphere. The coatings deposited on CoCrMo discs were used for mechanical and surface evaluation while the coatings on Si(001) wafers were used to evaluated cross-sectional morphology as well as XPS. During the depositions, the substrates faced a pure Si target (area 440 cm^2^, purity 99.8%) at a distance of 6 cm. All coatings were grown using an average cathode power and pulse frequency of 600 W and 200 Hz, respectively. A pulsed bias voltage of −100 V, synchronized to the cathode pulse, was applied to the substrate table. The investigated process parameters in Table 1 were the substrate temperature seen from the applied power to the heating elements and the nitrogen-to-argon flow ratio (*ƒ*_N_2_/Ar_), at a constant deposition pressure of 400 mPa. The heaters were set to 1 kW, 3 kW or 5 kW, corresponding to substrate temperatures of 200 °C, 350 °C and 430 °C, respectively. The *ƒ*_N_2_/Ar_ was set to 0.06, 0.17 and 0.30. Out of the nine possible combinations of coating parameters, the six processes that would result in the highest nitrogen contents were selected, the chosen combinations are presented in Table 1. For further details about the coating process the reader is referred to the paper by Schmidt et al. [23] where a similar setup is used. Six samples were coated in each process, except for process no. 4, where five samples were coated. Uncoated CoCrMo discs polished to a medical grade were used as reference.

### 4.2. Methods

#### 4.2.1. X-ray Photoelectron Spectroscopy (XPS)

The chemical composition and bonding states present in the SiN*_x_* coatings were investigated by XPS as (Axis UltraDLD, Kratos Analytical, Manchester, UK) using monochromatic Al (Kα) X-ray radiation (hν = 1486.6 eV). The base pressure in the analysis chamber during acquisition was <1 × 10^−7^ Pa. Survey spectra as well as XPS core level spectra of the Si2p, Ar2p, N1s, C1s, and O1s regions were recorded on as-deposited samples and after sputter cleaning for 120 s with a 2 keV Ar^+^ ion beam. The Ar^+^ beam was rastered over an area of 3 × 3 mm^2^ at an incidence angle of 20°. Sputter cleaning was carried out to remove the surface oxygen layer and adventitious carbon following air exposure. The core level spectra recorded after Ar^+^ etching were used to determine the chemical composition of the SiN*_x_* coatings. For this work, a Shirley-type background was subtracted and elemental cross sections provided by Kratos Analytical were applied. Data obtained from as-deposited samples were used to investigate the chemical bond structure of the SiN*_x_*. The deconvolution of the core level spectra and subtraction of the Shirley-type background was performed with the CasaXPS software. All spectra were referenced to the C–C(H) bond at a binding energy of 285 eV. Core level spectra were fitted using a Voigt peak shape with the Lorentzian contribution not exceeding 30%. The full width at half maximum (FWHM) of all components was restricted to a maximum of 1.8 eV. The comparatively broad FWHM is necessary for some components due to the amorphous nature of the coatings. For the development of the peak fit model not only the here presented coatings but also sputter deposited Si, thermally grown SiO_2_ and Si_3_N_4_ deposited by CVD served as internal references. Automatic charge compensation was applied throughout the acquisition, owing to the increasingly electrical insulating nature of the SiN*_x_* coatings as the N content increases.

#### 4.2.2. Scanning Electron Microscopy (SEM)

Cross-sectional SEM (LEO 1550 Gemini, Zeiss, Jena, Germany) was performed on coatings deposited on Si(001) in order to assess the morphology as well as coating thickness and thus the deposition rate.

#### 4.2.3. Transmission Electron Microscopy (TEM)

Cross-sectional, electron transparent samples were prepared using conventional focused ion beam (FIB) milling procedures FIB SEM (NVision 40, Zeiss, Jena, Germany) equipped with a Schottky field emission gun. Firstly, a thin layer of tungsten was deposited over the site of interest to prevent ion beam damage from the bulk milling procedure. At 30 keV, trenches around the site were milled using Ga^+^ ions. The lamella was then attached to a micromanipulator, detached from the bulk sample, attached to a copper grid and milled to electron transparency with a 10 keV ion beam [25,32].

TEM (Titan 80-300LB, FEI, Eindhoven, The Netherlands) operated at 300 keV was used for imaging, generating electron diffraction patterns of the samples. High-angle annular dark-field scanning transmission electron microscopy (HAADF STEM) was used for imaging to visualize compositional contrast.

#### 4.2.4. Vertical Scanning Interferometry (VSI)

In order to confirm a low enough surface roughness for nanoindentation measurements VSI measurements were conducted (WYKO NT-110, Vecco, Germany). An area of 451 × 594 µm^2^ was obtained, using a 10× objective and a field of view of 1. On each sample five different areas were investigated [33]. From this the arithmetic mean surface roughness (*R*_a_), maximum height (*R*_t_) and the average distance between the highest peak and lowest valley based on five sampling heights (*R*_z_) were calculated with the Vision software.

#### 4.2.5. Nanoindentation

Nanoindentation was performed using an ultra-high resolution nanoindenter (UNHT, Anton Paar, Austria) equipped with a Berkovich tip. To determine an appropriate load a progressive multi-cycle measurement with 30 cycles was performed with loads ranging from 50 µN to 50 mN. Subsequent indentations were performed with a load of 20 mN, which corresponded to a penetration depth of approximately 190–330 nm. Thirty indentations were performed on each sample. Nano-hardness and Young’s modulus were determined using the Oliver-Pharr method [34]. The values were based on a Poisson’s ratio, ν, of 0.25 [35] and calculated using the software belonging to Anton Paar.

#### 4.2.6. Statistical Analysis

Statistical analysis of differences between coatings was performed using IBM SPSS Statistics v22, at α = 0.05, by an analysis of variance (ANOVA) with a following Scheffe’s post-hoc test. The roughness parameters of the coatings were compared to CoCrMo by an ANOVA followed by Dunnett’s test. For the other parameters an ANOVA with a following Scheffe’s post-hoc test was performed.

## 5. Conclusions 

For dense and amorphous SiN*_x_* coatings deposited with rHiPIMS, we found that an increasing *ƒ*_N_2_/Ar_, up to 0.3, resulted in an increased nitrogen content with no apparent effect on the microstructure. The increased nitrogen content resulted in coatings with a higher nano-hardness and elastic modulus of 26 GPa and 293 GPa, respectively, when deposited at *ƒ*_N_2_/Ar_ = 0.30. At a substrate temperature of 430 °C, XPS showed a decrease in the nitrogen content in the coatings, and nanoindentation showed a corresponding decrease in the mechanical properties, as compared to 200 °C and 350 °C.

## Figures and Tables

**Table 1:** Heating power, resulting substrate temperature, nitrogen-to-argon flow ratio (*ƒ*_N_2_/Ar_), growth rate and thickness as well as resulting composition (as determined by XPS) for the different processes.

Process No.	Heating (kW)	*T* (°C)	*ƒ*_N_2_/Ar_	Growth Rate (nm/s)	Coating Thickness (µm)	Si (at.%)	N (at.%)	O (at.%)
1	1	200	0.06	0.50	7.5	71.7	22.8	1.8
2	1	200	0.17	0.40	7.3	54.6	39.2	3.6
3	1	200	0.30	0.34	7.1	45.6	48.0	5.0
4	3	350	0.17	0.42	7.6	54.4	40.9	2.0
5	3	350	0.30	0.35	7.4	45.2	47.5	5.8
6	5	430	0.30	0.40	7.3	53.9	41.3	2.1

**Table 2:** Surface roughness, hardness, Young’s modulus, N/Si ratio and *H*/*E* ratio for processes 1–6 and the CoCrMo reference.

Process No./CoCrMo Reference	N/Si Ratio	*R*_a_ (nm)	*R*_z_ (nm)	*R*_t_ (nm)	*H* (GPa)	*E* (GPa)	*H*/*E* Ratio
1	0.32	12.5 ± 2.2	330	1430	12.1 ± 0.8	172.7 ± 7.5	0.070
2	0.72	10.8 ± 1.8	377	1460	17.9 ± 1.3	239.1 ± 12.6	0.075
3	1.05	12.5 ± 3.7	461	2180	23.7 ± 3.0	292.6 ± 18.1	0.081
4	0.75	11.4 ± 1.8	365	1750	18.7 ± 1.2	226.1 ± 9.1	0.083
5	1.05	15.1 ± 2.7	1272	4710	25.9 ± 2.1	285.5 ± 20.1	0.091
6	0.76	12.8 ± 4.5	964	3800	19.6 ± 2.0	222.0 ± 13.3	0.088
CoCrMo	-	10.7 ± 1.8	282	870	6.2 ± 0.4	293.1 ± 17.7	0.021
